# Enhanced joint energy transfer potential by the biarticular gastrocnemii muscles during perturbed walking

**DOI:** 10.1007/s00421-025-05727-z

**Published:** 2025-03-05

**Authors:** Christos Theodorakis, Sebastian Bohm, Gaspar Epro, Falk Mersmann, Julian Werth, Kiros Karamanidis, Adamantios Arampatzis

**Affiliations:** 1 Department of Training and Movement Sciences, Humboldt-Universität zu Berlin, Philippstr 13, Haus 11, 10115 Berlin, Germany; 2 Berlin School of Movement Science, Berlin, Germany; 3 School of Applied and Health Sciences, College of Health and Life Sciences, London South Bank University, London, UK; 4 Department of Sport Science, Faculty for Mathematics and Natural Sciences, University of Koblenz, Koblenz, Germany

**Keywords:** Unpredictable perturbations, Adapted perturbations, Tripping, Dropping, Coupling angle

## Abstract

Our objective was to explore how the potential for energy transfer between the ankle and knee joint via the biarticular gastrocnemii muscles is modulated during unpredictable and adapted trip-like and drop-like gait perturbations. Using kinematic parameters of the ankle and knee joints, the energy transfer potential between the two joints was determined as the fraction of contact time when the ankle and knee joint angles are in-phase. Additionally, the electromyographic activity of the gastrocnemius medialis and lateralis were captured during the drop-like perturbations. The energy transfer potential increased 1.6-fold in the trip-like and 2.5-fold in the drop-like perturbations compared to unperturbed walking, indicating a relevant involvement of biarticular mechanisms in maintaining body stability. The activation of the gastrocnemii was high (50–60% of a maximum voluntary contraction) in the phases of ankle-to-knee and knee-to-ankle joint energy transfer, which suggests a relevant contribution of biarticular mechanisms to the management of the body’s energy during the drop-like perturbations. Considering the similar ankle-to-knee joint energy transfer potential compared to unperturbed walking, the higher activation of the gastrocnemii muscles in the first 20% of the stance indicates a greater contribution of biarticular mechanisms to the absorption of body energy in the unpredictable perturbations.

## Introduction

Movement on irregular surfaces is associated with external mechanical perturbations, reduced gait stability and an increased risk of falls (Berg et al. 1997; Boonkhao et al. 2024; Li et al. 2006). Fast and effective neuromotor responses, as for example, rapid force development of the plantar flexor muscles, are functionally highly relevant to prevent a fall (Pijnappels, Bobbert, and Van Dieën 2005; Robinovitch et al. 2002; Brüll et al. 2024). Investigations on leg and joint mechanics provide evidence that the ankle joint plays a key role for body stability in many animals (Daley and Biewener 2006; Daley, Felix, and Biewener 2007) including humans (Golyski and Sawicki 2022; Dick, Punith, and Sawicki 2019). In human steady locomotion, the ankle joint produces between 41 and 45% of the necessary power and work during walking and between 58 and 67% during running (Arampatzis et al. 2000; Farris and Sawicki 2012; Schache, Brown, and Pandy 2015). In perturbed locomotion, the contribution of the ankle joint increases to 64% during slip-like perturbations (Golyski and Sawicki 2022) and 66 to 80% during drop-like perturbations (Dick, Punith, and Sawicki 2019). The human soleus, gastrocnemius medialis (GM) and gastrocnemius lateralis (GL) form the triceps surae muscle and operate together as ankle extensors to generate moments and produce power at the ankle joint during locomotion. The biarticular gastrocnemii muscles generate moments in both the ankle and knee joints and can, therefore, reallocate power and work between the two joints (Cleland 1867; Bobbert et al. 1986).

Although, theoretically, two monoarticular muscles can generate similar moments at the joints they span as one biarticular muscle, the latter can reduce the cost of neural control of the musculoskeletal system due to their ability to act simultaneously on two joints (Schumacher et al. 2020). The coactivation of adjacent monoarticular and biarticular muscles allows the monoarticular muscle to act at a joint that it does not span, minimizing the mechanical delay of the system in response to neural commands (Van Ingen Schenau 1989; Prilutsky and Zatsiorsky 1994). For example, the biarticular gastrocnemii muscles can transfer power and energy from the more proximal monoarticular vasti to the ankle joint and thus regulate the redistribution and transfer of mechanical power and energy between the ankle and knee joints to be effective at the joint where it is needed (Van Ingen Schenau 1989; Kharazi et al. 2023). During slow locomotion, such as walking, stability in humans and animals is mainly maintained by a proactive strategy based on predictive and anticipatory control by supraspinal brain centres (Patla 2003; Biewener and Daley 2007). However, during fast movements or after locomotor perturbations, biological systems rely on the interaction of local spinal sensorimotor pathways with intrinsic properties of the musculoskeletal system to reduce the need for higher level control (Biewener and Daley 2007; Daley 2018).

There is evidence for a critical role of the ankle joint in human locomotion and indications for a muscle-specific functional relevance of the plantar flexors during perturbations (Dick et al. 2021). Although the three muscles work synergistically, they have important differences in function, geometry and size. The Soleus, as monoarticular muscle, acts around the ankle joint, while the GM and GL are biarticular and act across the ankle and knee joints. The involvement of biarticular mechanisms during gait perturbations may increase the intrinsic stability of biological systems to counteract perturbations, reducing the effort of neural control and the mechanical delay of the neural commands driving the motor responses. The important contribution of the biarticular mechanisms of the gastrocnemii muscles to the mechanical power and work performed at the ankle joint during maximal vertical jumping (Bobbert et al. 1986; Prilutsky and Zatsiorsky 1994) and during accelerated sprint running (Jacobs, Bobbert, and Van Ingen Schenau 1996) has been reported in the past using predictions from musculoskeletal models. More recently, experimental data have demonstrated a relevant involvement of the biarticular mechanisms of the gastrocnemii muscles in managing the mechanical power and work required at the ankle joint during fast to maximal walking speeds (Kharazi et al. 2023) as well as submaximal running (Arampatzis et al. 2023). Yet, to our knowledge, no study has investigated the possible involvement of the biarticular mechanisms of the gastrocnemii muscles during perturbations, where fast reactive neuromotor responses are essential to maintain stability and prevent falls.

There are two separate mechanisms by which the biarticularity of the gastrocnemii muscles can influence the mechanical power and work at the ankle joint, independent of their own musculotendinous power and work production (Van Ingen Schenau 1989; Prilutsky, Herzog, and Leonard 1996; Junius et al. 2017). First, an energy transfer between the ankle and knee joints is possible when the mechanical powers of the gastrocnemii muscles at the two joints have opposite signs (Prilutsky, Herzog, and Leonard 1996; Arampatzis et al. 2023). Second, same signs in the mechanical power of the gastrocnemii muscles at the two joints indicate a simultaneous energy absorption or production of the gastrocnemii muscles at the ankle and knee joint (Prilutsky, Herzog, and Leonard 1996; Arampatzis et al. 2023), which leads to a redistribution of their musculotendinous power and work. The gastrocnemii muscles generate plantarflexion moments at the ankle joint and flexion moments at the knee joint. Therefore, during a synchronous knee extension and plantarflexion or during a knee flexion and dorsiflexion (i.e., in-phase fluctuations at the two joints) the mechanical power of the gastrocnemii muscles at the ankle and knee joints will have opposite signs (i.e., the possibility of knee-to-ankle or ankle-to-knee joint energy transfer via the gastrocnemii muscles). On the other hand, in the case of an anti-phase fluctuation (i.e., knee flexion and plantar flexion or knee extension and dorsiflexion) the mechanical power of the gastrocnemii muscles at the ankle and knee joints will have the same sign (i.e. the possibility of simultaneous energy production or absorption at the two joints via the gastrocnemii muscles). This shows that kinematic data in a form of ankle and knee joint angular changes can provide information about the possible involvement of biarticular mechanisms during locomotion.

In the current study, the lower leg kinematics were recorded during unpredictable (without experience) and adapted (with experience) trip-like and drop-like gait perturbations. In addition, the electromyographic (EMG) activity of the biarticular GM and GL muscles of the perturbed leg was measured during the drop-like perturbations. Using vector coding we calculated the coupling angles of the ankle and knee joint angles to determine the in-phase and anti-phase fluctuations of the ankle and knee joint angles. Finally, based on the coupling angle, we determined the energy transfer potential as the fraction of contact time when the ankle and knee joint angles were in-phase. The primary goal was to better comprehend how the potential for energy transfer between the ankle and knee joint via the biarticular gastrocnemii muscles is modulated during unpredictable and adapted walking perturbations. It was expected that the biarticular mechanisms for energy transfer between the ankle and knee joints via the gastrocnemii muscles would be more involved in perturbed than in unperturbed walking.

## Methods

### Experimental design

**Fig. 1:**
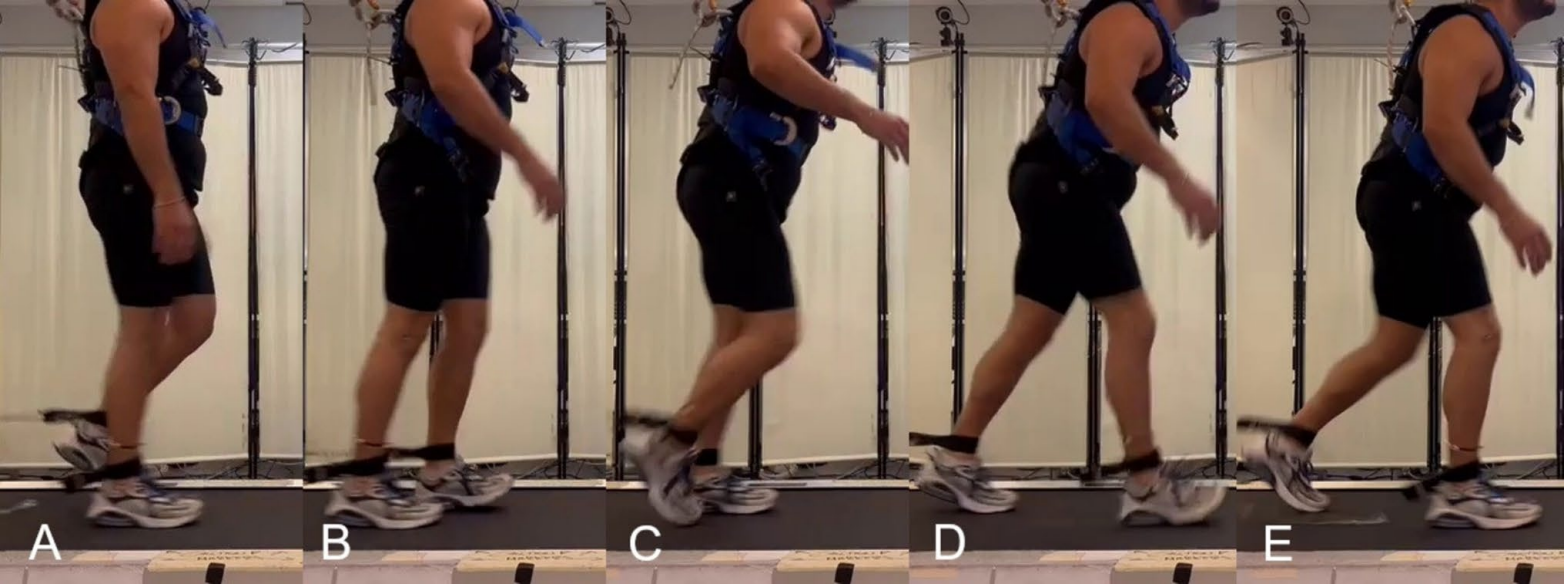
Characteristic time points during the trip-like perturbations. Initiation of the perturbation on the left (ipsilateral) leg by applying a force via an ankle strap while the right (contralateral) leg is in mid-stance (**A**), touch-down of the left leg after the perturbation (**B**), take-off of the right leg (**C**), touch-down of the right leg in the step after the perturbation (**D**), take-off of the left leg (**E**)

In two separate experiments, we investigated unpredictable (without experience) and adapted (repeated exposure with experience) trip-like and drop-like perturbations during walking. In the first experiment, twenty healthy individuals (age 27.3 ± 6.2 years, body mass 76.8 ± 14 kg, body height 176.9 ± 11.4 cm) were exposed to trip-like walking perturbations after giving informed consent. The experiment was approved by the ethics committee of the School of Applied Sciences at London South Bank University (SAS1826b) and conducted in accordance with the declaration of Helsinki. The perturbations were introduced while participants performed shod walking on a treadmill (Valiant 2 sport XL; Lode B.V., Groningen, The Netherlands) at 1.4 m/s (Fig. 1). The detailed description of the applied trip-like perturbations protocol can be found in an earlier report (Epro et al. 2018). Briefly, a gait perturbation was applied using a pneumatic brake and release system which applies a modulated force (~100 N) to the left shank via an ankle strap positioned right above the malleoli, attached by a Teflon cable to the perturbation device, in the direction opposite to movement. The force was applied during mid stance of the right leg and, thus, perturbed the motion of the left leg during mid-swing (the anterior velocity of the left malleolus became zero). The resistance was released at the touch-down of the left leg (Epro et al. 2018; Werth et al. 2022). The experimental protocol contained nine gait trials starting with one unperturbed level walking trial, followed by an unpredictable perturbation trial (no prior experience) and seven additional perturbation trials, in which the same perturbation occurred unpredictably, yet the participants’ response was adapted based on the prior experience. In all investigated perturbation trials, the participants had no knowledge of when their ankle would be pulled, but were aware that they would receive a perturbation at some point. For safety reasons, participants were strapped into a harness system to prevent the knees from touching the ground in case of a fall. In the analysis, we selected the unperturbed walking trial, the unpredictable perturbation trial and the last perturbation trial (i.e., adapted based on prior experience).

**Fig. 2:**
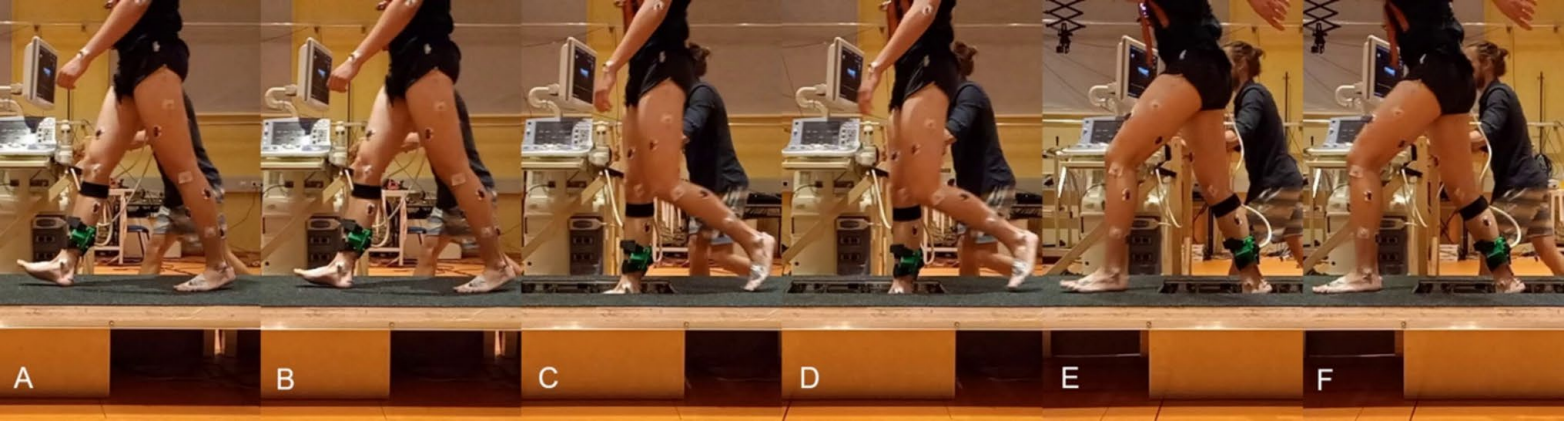
Characteristic time points during the drop-like perturbations. Touch-down of the right (ipsilateral) leg on the hidden dropping platform (**A**), time point of the surface drop (**B**), take-off of the left (contralateral) leg after surface drop (**C**), touch-down of the right leg in the hole after the 15 cm surface drop (**D**), touch-down of the left leg in the step after the perturbation (**E**), take-off of the right perturbed leg (**F**)

In the second experiment, 26 healthy individuals (age 25.8 ± 5.4 years, body mass 73.8 ± 9.9 kg, body height 176.0 ± 7.3 cm) participated in the study of drop-like perturbations (15 cm drop of the support surface) after signing informed consent. The experiment was approved by the ethics committee of the Humboldt-Universität zu Berlin (HU-KSBF-EK_2018_0005) and conducted in accordance with the Declaration of Helsinki. The unpredictable and predictable drop-like perturbations were introduced during walking at preferred speed (1.3 ± 0.3 m/s), using a hidden movable platform with an electronically triggered dropping plate (70 × 46 × 15 cm^3^) mounted in the second half of an 18-m-long walkway (Fig. 2). The starting position at the beginning of the walkway was adjusted during familiarization trials to ensure that participants always contacted the platform with their right leg (perturbed leg). The perturbations were introduced by releasing the electronic dropping plate in the beginning of the double contact phase (~30 ms after touch-down of the right leg). The release of the plate was triggered when the calcaneus marker passed a spatial threshold in the walking direction. The experimental protocol was comprised of ten gait trials starting with three unperturbed level walking trials, followed by an unpredictable drop-like gait perturbation (without prior experience) and six predictable perturbation trials, in which the plate-release was announced and the participants adapted their gait based on the prior experience. Even though the participants were informed that they would receive some sort of perturbation to their gait in the unpredictable perturbation, they had no knowledge of the perturbation mechanism and the kind of perturbation. On the other hand, in the six predictable perturbation trials, the participants could clearly see the location of the dropping-plate and were instructed that the perturbation would occur when placing their right leg on the platform. To prevent injury in case of a fall, the participants were secured via a harness that was mounted to the ceiling and could freely follow them along the walkway. In the analysis, we included the first unperturbed walking trial, the unpredictable perturbation trial and the last predictable perturbation trial (i.e., adapted based on prior experience).

### Measurement of joint kinematics and electromyographic activity

Lower leg kinematics were recorded at 120 Hz for the trip-like (Qualisys AB, Göteborg, Sweden) and 250 Hz for the drop-like perturbations (Vicon Motion Systems, Oxford, UK). Reflective markers (14 mm in diameter) were attached on anatomical landmarks of the left leg during the trip-like perturbations and the right leg during the drop-like perturbation: the tip of the second metatarsal, lateral malleolus, the notch of the tuber calcaneus, lateral epicondyle and the greater trochanter. The 3D-coordinates of the markers were filtered using a fourth-order low pass and zero-phase shift Butterworth filter with a cut-off frequency of 12 Hz. Ankle and knee joint angles were calculated in the sagittal plane and defined as the angle between the tip of the second metatarsal, lateral maleolus and lateral epicondyle for the ankle and lateral maleolus, lateral epicondyle and greater trochanter for the knee. The 0° ankle angle and 180° knee angle were defined based on the participant’s posture during quiet standing. An ankle dorsiflexion is represented by a decrease of ankle angle while an ankle plantar flexion by an increase. At the knee joint, a knee flexion is represented by a decrease of knee angle while a knee extension by an increase.

During the drop-like perturbations, a wireless system (Myon m 320RX, Myon AG, Baar, Switzerland) was used to measure the EMG activity of the biarticular GM and GL muscles of the right leg. The EMG activity was recorded at a sampling frequency of 2000 Hz. A fourth-order high-pass zero-phase shift Butterworth filter with a 20-Hz cut-off frequency, a full wave rectification and a low-pass zero-phase shift filter with a 20-Hz cut-off frequency was applied to the raw EMG signals. The EMG activity of the GM and GL were normalized to the highest measured EMG value recorded during a maximum plantar flexion voluntary contraction (MVC) at an ankle joint angle of 0°. The normalized EMG activities $\left(\hat{u} \right)$ of the two gastrocnemii were processed with the first order differential equation proposed by Zajac (Zajac 1989) to estimate their activation $\hat{\alpha }$ (Eq. 1).

1 $$\frac{{d\hat{\alpha }\left( t \right)}}{dt} + \left( {\frac{1}{{\tau_{act} }}\left( {\beta + \left( {1 + \beta } \right)\hat{u}\left( t \right)} \right)} \right)\hat{\alpha }\left( t \right) = \left( {\frac{1}{{\tau_{act} }}} \right)\hat{u}\left( t \right)$$

The time activation constant $\left({\tau }_{act}\right)$ and the ratios of the activation to deactivation time constant $\left(\beta \right)$ were taken from Dick et al., (Dick, Biewener, and Wakeling 2017) assuming a fibre distribution of 50% of type I and 50% of type 2 fibres for both gastrocnemii (Polgar et al. 1973; Edgerton, Smith, and Simpson 1975). Finally, we calculated an average weighted activation of the gastrocnemii muscles according to the ratios of their physiological cross-sectional areas (i.e., 2/3 GM and 1/3 GL; taken from Albracht et al., (Albracht, Arampatzis, and Baltzopoulos 2008)).

### Assessment of biarticular mechanisms

Using vector coding (Needham, Naemi, and Chockalingam 2014), we calculated the coupling angle (γ) of the ankle and knee joint angles to determine the in-phase and anti-phase fluctuations of the ankle and knee joint angles during the stance phase of the perturbed leg (left for the trip-like and right for the drop-like perturbations). For each percentage of the normalized stance phase of the unperturbed and perturbed gait trials, the coupling angle was calculated from the proximal (knee) and distal (ankle) joint angles using the equations reported by Needham et al., (Needham, Naemi, and Chockalingam 2014). The average coupling angle was calculated at each percentage of the stance phase using circular statistics (Batschelet 1981; Hamill et al. 1999). A coupling angle of 0° ≤ γ < 90° indicates in-phase fluctuations between ankle and knee joint angles (i.e., both increase) and a possibility of a knee-to-angle joint energy transfer via the biarticular gastrocnemii muscles. A coupling angle of 90° ≤ γ < 180° indicates anti-phase fluctuations between the two angles with a decrease in the knee joint angle and an increase in the ankle joint angle. In this phase, the gastrocnemii muscles have the possibility to produce energy in both the ankle and knee joint (simultaneous energy production). A coupling angle of 180° ≤ γ < 270° indicates again in-phase fluctuations between ankle and knee joint angles (i.e., both decrease) and a possibility of an ankle-to-knee joint energy transfer via the biarticular gastrocnemii muscles. Finally, a coupling angle of 270° ≤ γ < 360° indicates anti-phase fluctuations between the two angles (i.e., increase knee joint angle and decrease ankle joint angle) and a possibility of a simultaneous energy absorption in the two joints via the biarticular gastrocnemii muscles.

Based on the coupling angles during the stance phase, it is possible to determine the fraction of the contact time where the biarticular gastrocnemii muscles may transfer energy from ankle-to-knee joint and vice versa or to simultaneously absorb or produce energy at the two joints. Subsequently, we defined the fraction of the contact time that the ankle and knee joint angles are in-phase as energy transfer potential. Finally, the fraction of the contact time where energy can be transferred from knee-to-ankle joint (i.e., 0° ≤ γ < 90°) was defined as knee-to-ankle joint energy transfer potential and the fraction of contact time where energy can be transferred from ankle-to-knee joint (i.e., 180° ≤ γ < 270°) as ankle-to-knee joint energy transfer potential.

### Statistics

Our analysis included one stance phase for each trial of every participant (i.e., temporal and spatial walking parameters, kinematics, EMG, coupling angle, energy transfer potential). A linear mixed model, in the style of a one-way ANOVA, was used to test for the main effect of perturbation condition (unperturbed, unpredictable, and adapted) on the investigated outcomes and was applied separately on the two perturbation types (trip-like and drop-like). For the linear mixed model, the participants were treated as random effects and perturbation condition as fixed effect. In the case of a significant main effect of perturbation condition, pairwise comparisons were performed as a post-hoc analysis and Benjamini–Hochberg corrected p-values are reported. We conducted the statistical analyses using R v4.0.1 (R Foundation for Statistical Computing Vienna, Austria), and the ’nlme’ and ’emmeans’ packages were used for the linear mixed model and post-hoc testing, respectively. The significance level was set to α = 0.05.

## Results

**Fig. 3:**
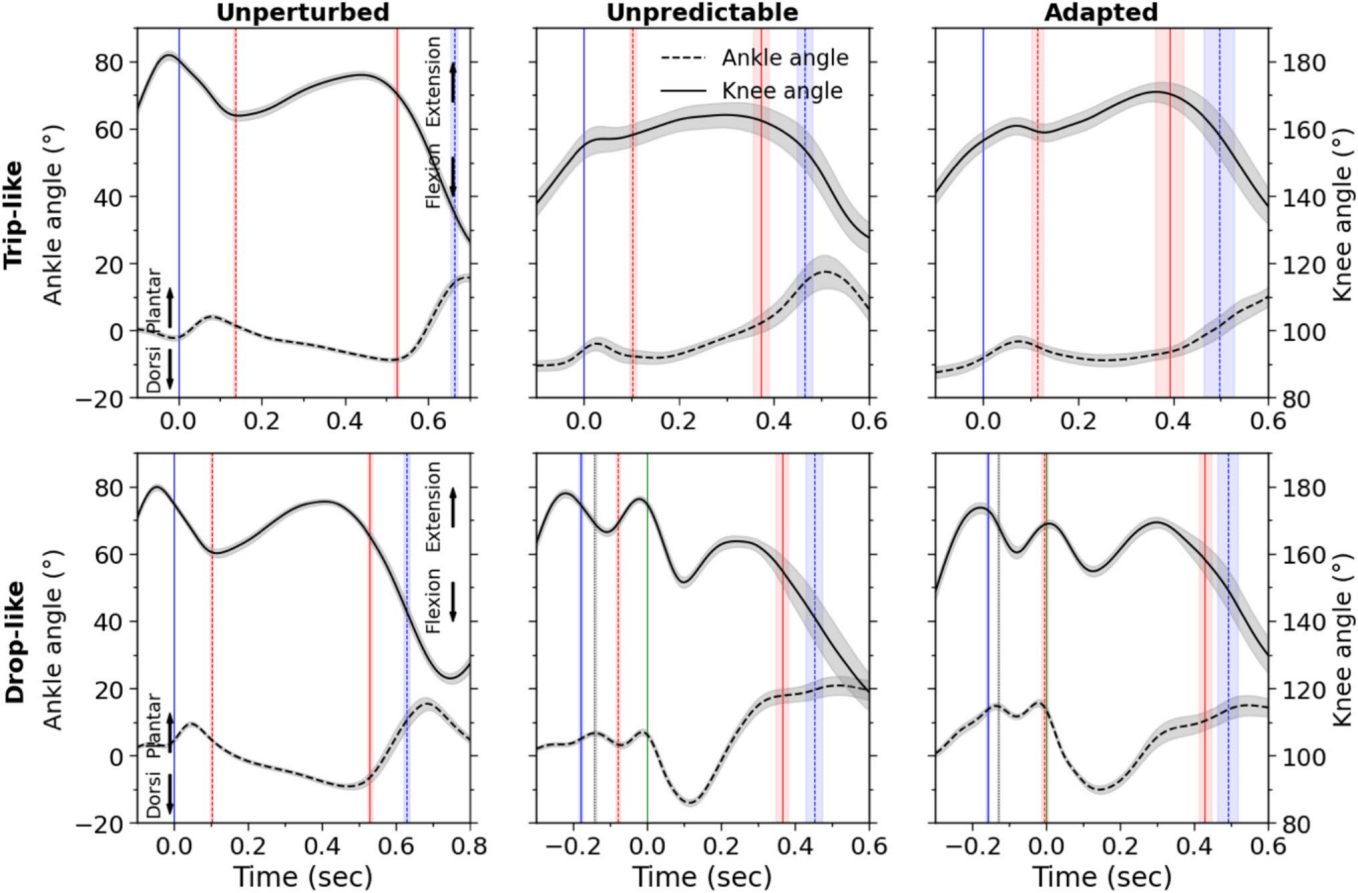
Ankle and knee joint angles during unperturbed walking, unpredictable without prior experience and adapted with experience walking perturbations for the trip-like and drop-like perturbation experiments. The vertical solid lines show the touch-down of the ipsilateral (blue) and contralateral (red) leg, the vertical dashed lines indicate the take-off of the contralateral (red) and ipsilateral (blue) leg. In the drop-like perturbations the vertical dotted black lines display the time point of the surface drop (15 cm) of the movable platform in the unpredictable and adapted perturbations and the vertical green solid lines (zero in the horizontal axis) show the touch-down of the ipsilateral leg after the 15 cm surface drop in the unpredictable and adapted perturbation trials. Note that the ankle joint angle is presented in the left vertical axis and the knee joint angle in the right vertical axis. The curves/lines and shaded areas represent mean ±standard errors (*n* = 20 for the trip-like and n=26 for the drop-like perturbations)

Stance time decreased significantly (*p*<0.001) in the unpredictable and adapted perturbation trials compared to unperturbed walking for both trip-like and drop-like perturbations (Table 1). There were no significant differences in the stance time between the unpredictable and adapted perturbations (*p* = 0.358 for the trip-like and *p* = 0.209 for the drop-like perturbations). In the unpredictable and adapted trip-like perturbations, the participants showed a more dorsiflexed ankle joint (*p*<0.001) and a more flexed knee joint (*p*<0.001) at touch-down compared to unperturbed walking (Table 1 and Fig. 3), with no differences between the unpredictable and adapted trip-like perturbation trials (*p* = 0.160 for the ankle and *p* = 0.773 for the knee joint angle). In the drop-like perturbations, the ankle and knee joint angles did not differ between the unperturbed and unpredictable trials at touch-down before the drop of the plate (*p* = 0.552 for the ankle and p = 0.870 for the knee joint). However, in the adapted perturbations, the ankle joint was more plantarflexed (*p*<0.001) compared to the unperturbed walking and unpredictable perturbations (Table 1 and Fig. 3). The participants in the adapted drop-like perturbations showed a more plantarflexed ankle (*p*<0.001) and a more flexed knee joint (*p*<0.001) at touch-down after the drop of the plate compared to the unpredictable perturbations (Table 1 and Fig. 3).

**Table 1:** Time duration of the stance phase and joint angles during unperturbed walking (unperturbed), unpredictable without experience (unpredictable) and adapted walking perturbations (adapted) in the trip-like and drop-like perturbation experiments

	Unperturbed	Unpredictable	Adapted
Trip-like perturbation experiment			
Stance time (ms)	663 ± 37 **a**	466 ± 73 **b**	496 ± 140 **b**
Ankle angle at touch-down (°)	-1.7 ± 3.7 **a**	-5.7 ± 5.8 **b**	-7.1 ± 5.1 **b**
Knee angle at touch-down (°)	179.5 ± 5.6 **a**	154.7 ± 9.4 **b**	155.1 ± 10.0 **b**
Drop-like perturbation experiment			
Stance time (ms)	630 ± 43 **a**	452 ± 105 **b**	490 ± 134 **b**
Ankle angle at touch-down (°)	4.6 ± 3.2 **a**	4.7 ± 2.7 **a**	13.7 ± 6.0 **b**
Knee angle at touch-down (°)	174.7 ± 4.7 **a**	174.7 ± 4.2 **a**	173.5 ± 7.7 **b**
Ankle angle at touch-down after drop (°)		5.9 ± 4.8 **a**	12.9 ± 6.1 **b**
Knee angle at touch-down after drop (°)		174.4 ± 6.2 **a**	168.9 ± 5.8 **b**

Note that negative values of the ankle joint angles indicate dorsiflexion and positive values plantar flexion. The values in a row that share the same letter (a or b) do not differ significantly (*p* > 0.05, post hoc analysis)

**Fig. 4:**
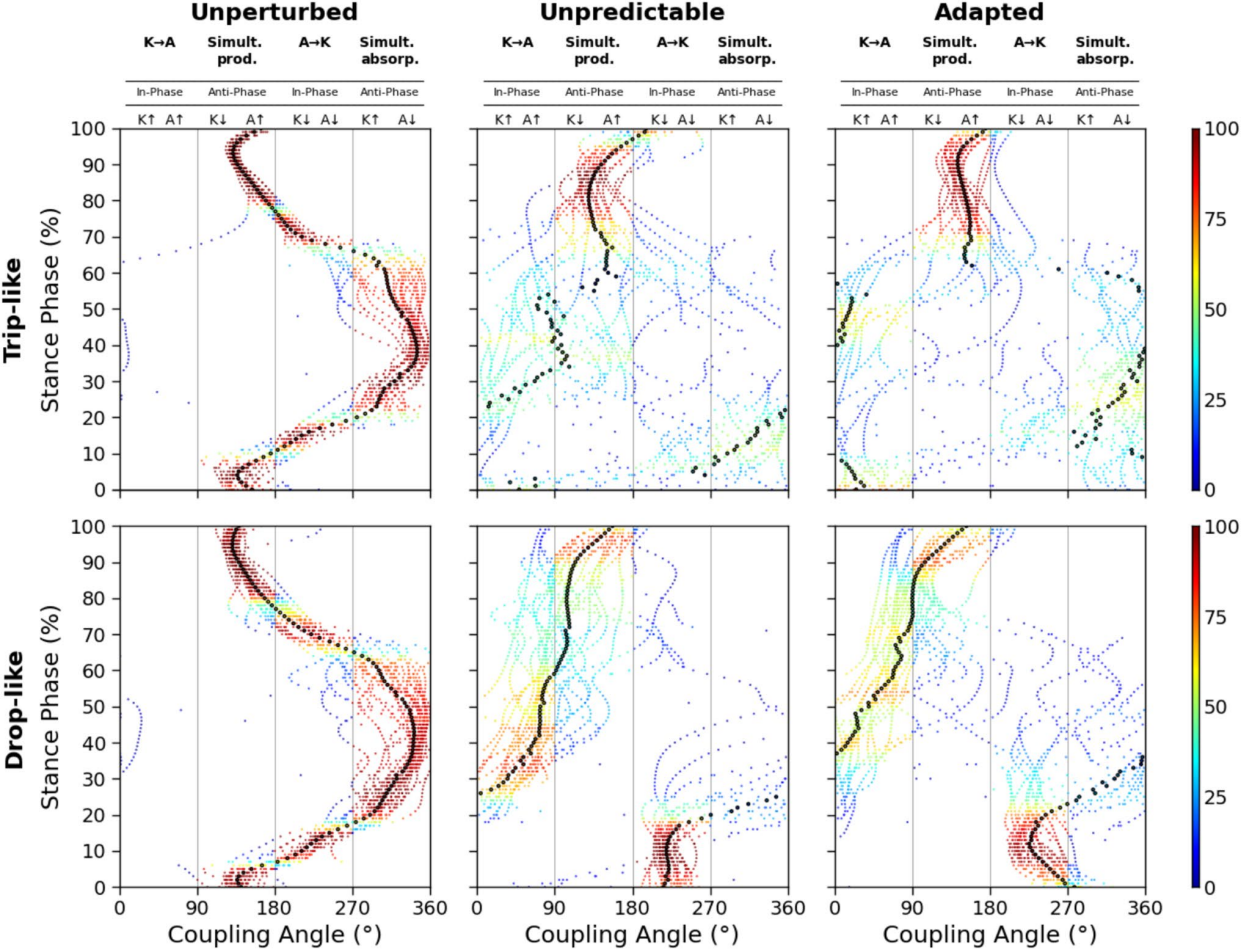
Average (large circles) and individual (small circles) coupling angles (γ) of the ankle and knee joint angles in the time-normalized stance phase (vertical axis) of the ipsilateral leg during unperturbed walking, unpredictable without prior experience and adapted with experience walking perturbations for the trip-like and drop-like perturbation experiments. 0° < γ ≤ 90^o^ indicates in-phase fluctuations with increasing of knee (K↑) and ankle (A↑) joint angles and potential for knee-to-ankle joint energy transfer (K→A) via the biarticular gastrocnemii muscles, 90° < γ ≤ 180° indicates anti-phase fluctuations with decreasing of knee (K↓) and increasing ankle (A↑) joint angles and potential for simultaneous energy production (Simult. prod.) at the ankle and knee joint via the biarticular gastrocnemii muscles, 180° < γ ≤ 270° indicates in-phase fluctuations with decreasing of knee (K↓) and ankle (A↓) joint angles and potential for ankle-to-knee joint energy transfer (A→K) via the biarticular gastrocnemii muscles, 270° < γ ≤ 360° indicates anti-phase fluctuations with increasing of knee (K↑) and decreasing ankle (A↓) joint angles and potential for simultaneous energy absorption (Simult. absorb.) at the ankle and knee joint via the biarticular gastrocnemii muscles. The color of the circles represents the relative frequency of the participants in each of the four phases for every percentage of the stance phase

**Fig. 5:**
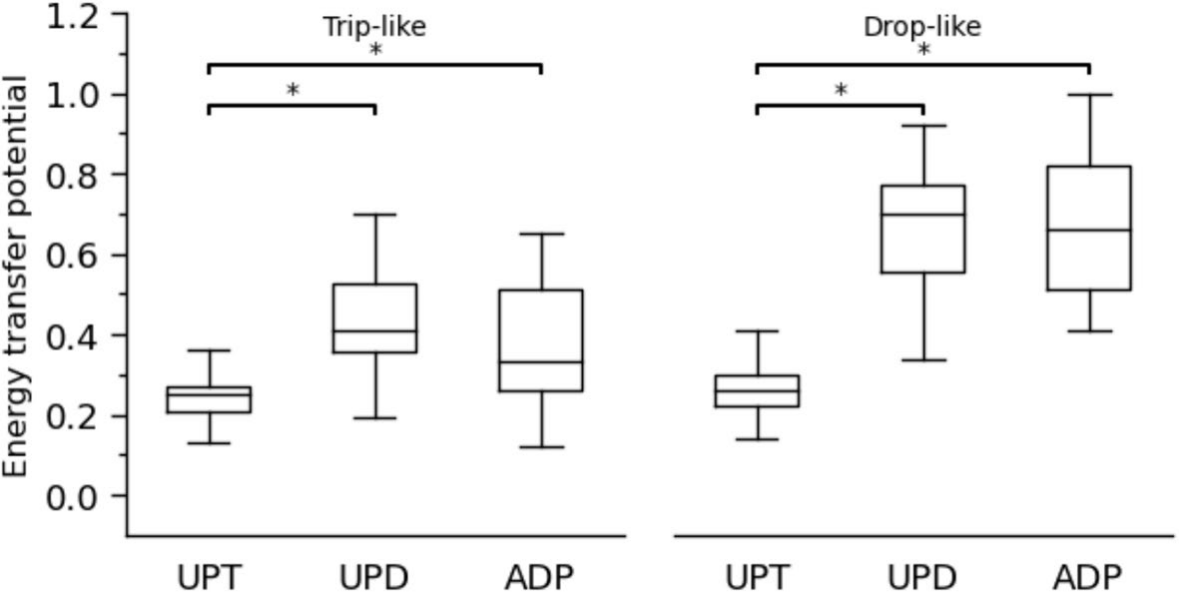
Energy transfer potential via the biarticular gastrocnemii muscles during the stance phase in unperturbed walking (UPT), unpredictable without prior experience (UPD) and adapted with experience (ADP) walking perturbations for the trip-like and drop-like perturbation experiments. * Statistically significant (p<0.05) differences

**Fig. 6:**
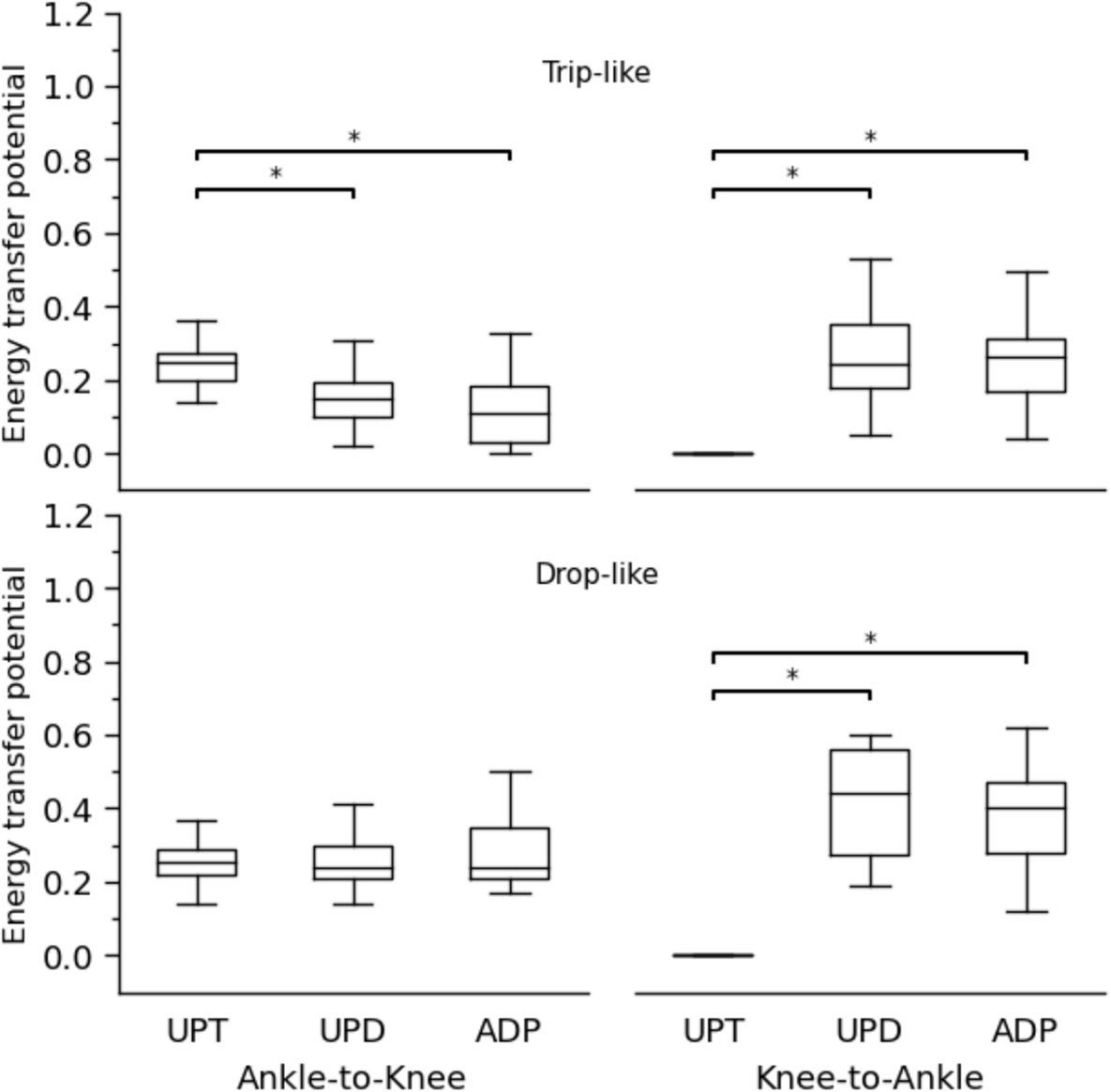
Ankle-to-knee joint and knee-to-ankle joint energy transfer potential via the biarticular gastrocnemii muscles during the stance phase in unperturbed walking (UPT), unpredictable without prior experience (UPD) and adapted with experience (ADP) walking perturbations for the trip-like and drop-like perturbation experiments. * Statistically significant (*p*<0.05) differences

Concerning the coupling angles and the determined in-phase and anti-phase fluctuations of the two analysed joints, a clear shift from simultaneous energy absorption during unperturbed walking to energy transfer from the knee to the ankle joint via the biarticular gastrocnemii was detected in the perturbed conditions (Fig. 4). There was a significant increase of the energy transfer potential for both trip-like (*p*<0.001) and drop-like (*p*<0.001) perturbations in the perturbed compared to the unperturbed walking (Fig. 5). The increase in the energy transfer potential was 1.6-fold in the trip-like and 2.5-fold in the drop-like perturbations. The energy transfer potential did not differ between the unpredictable and adapted walking perturbations (*p* = 0.486 for the trip-like and *p* = 0.956 for the drop-like perturbations, Fig. 5). More specifically, the potential of the knee-to-ankle joint energy transfer increased significantly for both, trip-like (*p*<0.001) and drop-like (*p*<0.001) perturbations compared to the unperturbed walking (Fig. 6). However, there were no significant differences between unpredictable and adapted perturbations (*p* = 0.829 for the trip-like and *p*=0.496 for the drop-like perturbations). The potential of ankle-to-knee joint energy transfer decreased (*p*<0.001) during the trip-like perturbations compared to unperturbed walking, but remained unchanged (*p* = 0.214 to *p* = 0.937) during the drop-like perturbations in all gait conditions (Fig. 6).

**Fig. 7:**
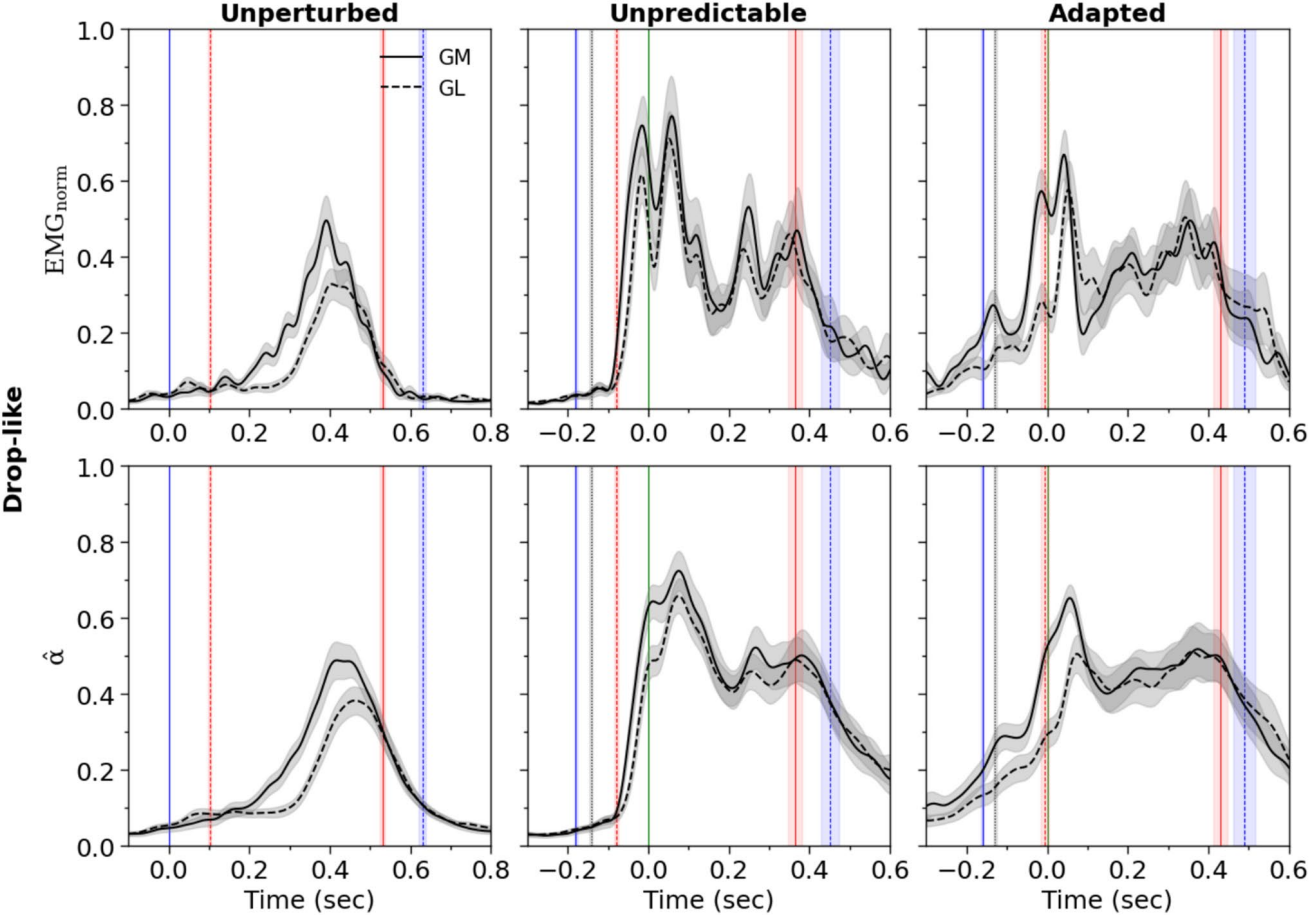
Normalized to maximum voluntary contraction electromyographic activity (EMG_norm_) and activation $\hat{\alpha }$^ of the gastrocnemius medialis (GM) and gastrocnemius lateralis (GL) muscles during unperturbed walking, unpredictable without prior experience and adapted with experience walking perturbations in the drop-like perturbation experiments. The vertical solid lines show the touch-down of the ipsilateral (blue) and contralateral (red) leg, the vertical dashed lines indicate the take-off of the contralateral (red) and ipsilateral (blue) leg, the vertical dotted black lines display the time point of the surface drop (15 cm) of the movable platform in the unpredictable and adapted perturbations and the vertical green solid lines (zero in the horizontal axis) show the touch-down of the ipsilateral leg after the 15 cm surface drop in the unpredictable and adapted perturbation trials. The curves/lines and shaded areas represent mean ±standard errors (*n* = 26)

**Fig. 8:**
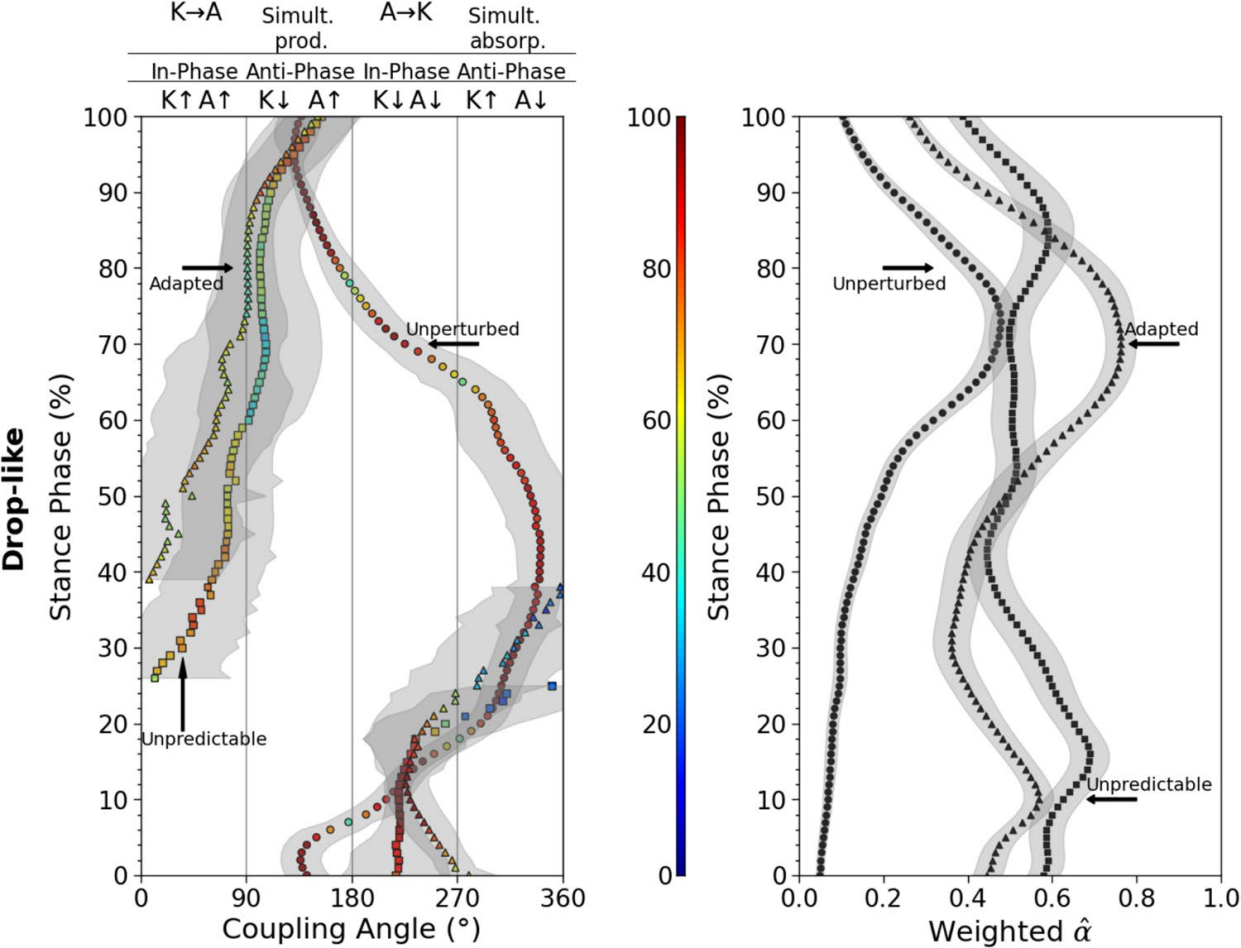
Coupling angles (γ) of the ankle and knee joint angles and weighted activation (Weighted $\hat{\alpha }$^) of the gastrocnemius medialis and gastrocnemius lateralis muscles during unperturbed walking, unpredictable without prior experience and adapted with experience walking perturbations in the drop-like perturbation experiments. 0° < γ ≤ 90° indicates in-phase fluctuations with increasing of knee (K↑) and ankle (A↑) joint angles and potential for knee-to-ankle energy transfer (K→A) via the biarticular gastrocnemii muscles, 90° < γ ≤ 180° indicates anti-phase fluctuations with decreasing of knee (K↓) and increasing ankle (A↑) joint angles and potential for simultaneous energy production (Simult. prod.) at the ankle and knee joint via the biarticular gastrocnemii muscles, 180° < γ ≤ 270° indicates in-phase fluctuations with decreasing of knee (K↓) and ankle (A↓) joint angles and potential for ankle-to-knee joint energy transfer (A→K) via the biarticular gastrocnemii muscles, 270° < γ ≤ 360° indicates anti-phase fluctuations with increasing of knee (K↑) and decreasing ankle (A↓) joint angles and potential for simultaneous energy absorption (Simult. absorb.) at the ankle and knee joint via the biarticular gastrocnemii muscles. The color of the circles represents the relative frequency of the participants in each of the four phases for every percentage of the stance phase. The curves represent mean ±variability for the coupling angles and mean ±standard errors for the weighted activation (*n* = 26)

**Fig. 9:**
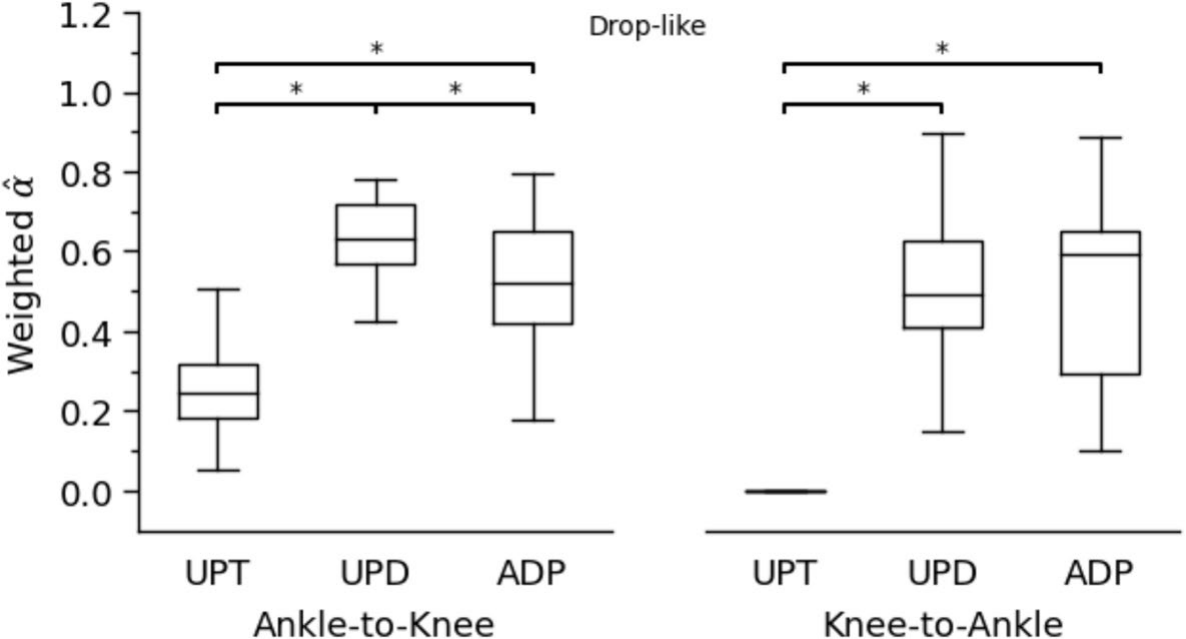
Weighted activation of the gastrocnemius medialis and gastrocnemius lateralis muscles in the phase of ankle-to-knee and knee-to-ankle joint energy transfer during the stance phase in unperturbed walking (UPT), unpredictable without prior experience (UPD) and adapted (ADP) with experience walking perturbations for the drop-like perturbation experiments. * Statistically significant (*p*<0.05) differences

In the drop-like perturbations, both the maximum as well as average normalized EMG activity and activation of the GM and GL muscles during the stance phase increased significantly (*p*<0.001) in both unpredictable and adapted perturbations compared to unperturbed walking (Fig. 7 and Table 2). There were no significant differences in the maximum and average EMG activity and activation of the two muscles between the unpredictable and adapted gait perturbations (*p* = 0.356 to *p* = 0.963, Table 2). The average weighted activation of GM and GL during the ankle-to-knee and knee-to-ankle joint energy transfer phases increased significantly (*p*<0.001) during both unpredictable and adapted walking perturbations compared to unperturbed walking (Fig. 8 and 9), with no differences between unpredictable and adapted perturbations during the knee-to-ankle joint energy transfer phase (*p* = 0.993). However, a significantly higher average weighted activation of these muscles was found during the ankle-to-knee joint energy transfer phase (*p* = 0.017) of the unpredictable walking perturbations (Fig. 9).

**Table 2:** Normalized to maximum voluntary contraction, maximum (max) and average (avg) electromyographic activity (EMG_norm_) and activation of the gastrocnemius medialis (GM) and lateralis (GL) muscles of the perturbed leg during the stance phase of the unperturbed walking (unperturbed), unpredictable without prior experience (unpredictable) and adapted with experience walking perturbations (adapted) in the drop-like perturbation experiment

	Unperturbed	Unpredictable	Adapted
EMG_GM,max_ (norm)	0.65 ± 0.31 **a**	1.25 ± 0.61 **b**	1.05 ± 0.29 **b**
EMG_GL,max_ (norm)	0.51 ± 0.28 **a**	1.13 ± 0.42 **b**	1.21 ± 0.53 **b**
Activation_GM,max_	0.58 ± 0.22 **a**	0.89 ± 0.28 **b**	0.85 ± 0.17 **b**
Activation_GL,max_	0.46 ± 0.21 **a**	0.82 ± 0.24 **b**	0.89 ± 0.27 **b**
EMG_GM,avg_ (norm)	0.17 ± 0.09 **a**	0.47 ± 0.23 **b**	0.41 ± 0.12 **b**
EMG_GL,avg_ (norm)	0.12 ± 0.07 **a**	0.39 ± 0.15 **b**	0.41 ± 0.19 **b**
Activation_GM,avg_	0.22 ± 0.10** a**	0.56 ± 0.19 **b**	0.53 ± 0.13 **b**
Activation_GL,avg_	0.17 ± 0.08** a**	0.50 ± 0.15 **b**	0.49 ± 0.18 **b**

The values in a row that share the same letter (a or b) do not differ significantly (*p* > 0.05, post hoc analysis)

## Discussion

The current study investigated how the potential for energy transfer between the ankle and knee joint via the biarticular gastrocnemii muscles is modulated during unpredictable and adapted trip-like and drop-like gait perturbations using kinematic parameters of the ankle and knee joints. In line with our hypothesis we found a significant increase in the energy transfer potential, particularly the potential for energy transfer from the knee to the ankle joint, in both trip-like and drop-like perturbations compared to unperturbed walking. The increased energy transfer potential indicates a relevant involvement of biarticular mechanisms in maintaining body stability after the gait perturbations. Furthermore, the high activation of the gastrocnemii muscles in both the ankle-to-knee and knee-to-ankle joint energy transfer phases suggests a relevant contribution of biarticular mechanisms to the management of the body's energy during the drop-like perturbations. The higher activation of the gastrocnemii muscles in the ankle-to-knee joint energy transfer phase in combination with the unchanged ankle-to-knee joint energy transfer potential indicates a greater involvement of biarticular mechanisms during the unpredictable compared to the adapted drop-like walking perturbations.

As previously reported (Hak et al. 2012; AminiAghdam et al. 2019; Brüll et al. 2024), both trip-like and drop-like perturbations reduced contact time by 25–30% compared to unperturbed walking. Following a gait perturbation, rapid motor responses are essential to maintain stability and avoid falling (Karamanidis et al. 2020; Pai et al. 2010). Humans require four to five recovery steps to return to the original periodic pattern after a gait perturbation (Epro et al. 2018; Brüll et al. 2024). Therefore, the execution of multiple fast steps has been interpreted as a mechanism to increase the safety features of the system (Reimann, Fettrow, and Jeka 2018). Regarding this issue, the more dorsiflexed ankle joint and more flexed knee joint at touch-down after the trip-like perturbations indicate a more effective lower leg configuration for joint moment generation, i.e. closer to the optimal joint angles for moment generation compared to unperturbed walking (Herzog, Abrahamse, and ter Keurs 1990; Herzog, Read, and ter Keurs 1991). Rapid torque development following a tripping perturbation is functionally relevant to prevent falling (Pijnappels, Bobbert, and Van Dieën 2005). A rapid recovery step to increase the base of support has been also reported to be very important for the dynamic stability of the body in both young and old participants after unpredictable drop-like perturbations (Bierbaum et al. 2011; Bierbaum, Peper, and Arampatzis 2013). It is important to note that the increased plantarflexion angle at touch-down after the plate drop is an important stabilising strategy after drop-like perturbations (Van Der Linden et al. 2007; Müller, Ernst, and Blickhan 2012), which contributes to the necessary absorption of the body’s kinetic energy in the first part of the stance phase (Van Dieën et al. 2007). In this context, the changes found in leg kinematics (i.e., greater plantar flexion and knee flexion) at touch-down prior to the plate-drop in the predictable drop-like perturbations are evidence of experience-based proactive adjustments in motor control to prepare for the expected perturbation.

The main and most innovative finding of the current study is the increased potential for energy transfer between the ankle and knee joint via the biarticular gastrocnemii muscles during both trip-like and drop-like perturbations. The increase in energy transfer potential compared to unperturbed walking was 1.6-fold and 2.5-fold for the trip-like and drop-like perturbations, respectively. This increased energy transfer potential via the gastrocnemii muscles during perturbed walking demonstrates a crucial modulation of biarticular mechanisms to counteract locomotor perturbations. Furthermore, the results show that in both perturbation types an increase in the knee-to-ankle joint energy transfer potential was the reason for the increased potential of energy transfer between the two joints. The synchronous knee extension and plantar flexion (in-phase fluctuations) allows mechanical work and power to be effectively transferred to the ankle joint via the biarticular gastrocnemii from the monoarticular vasti muscles (Cleland 1867; Bobbert et al. 1986; Van Ingen Schenau 1989), which are more voluminous than the plantar flexors (Mersmann et al. 2014; Mersmann et al. 2015). Several studies in the past have reported the critical role of the ankle joint and the plantar flexor muscles in controlling body stability and producing the mechanical power and work required in challenging, perturbed locomotor conditions (Nakazawa et al. 2004; Van Dieën et al. 2007; Dick, Punith, and Sawicki 2019). The increased knee-to-ankle joint energy transfer potential implies a possible contribution of the vasti muscles, due to the biarticularity of the gastrocnemii, to the needed mechanical power and work at the ankle joint. The more voluminous vasti muscles (Falk Mersmann et al. 2014; F. Mersmann et al. 2015) can produce more muscular power and work than the distal triceps surae muscles. On the other hand, their longer fibres compared to the shorter triceps surae muscles increase the metabolic energy per unit of force generation (Biewener, Konieczynski, and Baudinette 1998) and thus the knee-to-angle joint energy transfer mechanism may increase the metabolic cost of locomotion. Consistent with this, Kharazi et al., (Kharazi et al. 2023) reported a negligible magnitude of energy transfer from the knee to the ankle joint at preferred walking speeds, with a significant increase at maximal walking speeds, where the metabolic energy cost is even higher than running (Minetti, Ardigò, and Saibene 1994). The applied trip-like and drop-like gait perturbations challenged the stability of the participants and increased the risk of falling. During the required fast reactive responses-possibly executed with low involvement of the corticospinal system (Biewener and Daley 2007)-mechanisms responsible for gait stability (i.e., the knee-to-angle joint energy transfer mechanism) may be prioritized over metabolic costs.

We found a perturbation type-specificity in the ankle-to-knee joint energy transfer potential, i.e. the ankle-to-knee joint energy transfer potential decreased during the trip-like perturbations compared to normal walking, but remained unchanged during the drop-like perturbations. This may be related to the task-specific requirements to manage the mechanical energy of the centre of mass after the perturbation. After trip-like walking perturbations, the ankle joint performs more positive than absorbing work during the stance phase (Shokouhi, Mokhtarzadeh, and Lee 2023) and, therefore, the involvement of biarticular mechanism of the gastrocnemii for energy absorption may not be relevant. On the other hand, in the first part of the stance phase following drop-like perturbations, the ankle contributes significantly to the absorption of the total energy of the centre of mass and decelerates the downward movement of the body (Van Dieën et al. 2007). Therefore, energy transfer from the ankle to the knee joint via the biarticular gastrocnemii muscles facilitates ankle joint energy absorption (Prilutsky and Zatsiorsky 1994; Arampatzis et al. 2023). During the first 20% of the stance phase of both unpredictable and adapted drop-like walking perturbations, the ankle and knee joint angles decrease, indicating a potential for ankle-to-knee joint energy transfer via the biarticular gastrocnemii muscles (Fig. 8). In the same time interval, the weighted activation of the gastrocnemii muscles is quite high (on average 50–60% of maximum). This high activation of the gastrocnemii muscles in the phase of energy transfer from the ankle to the knee joint during the drop-like perturbations indicates active force generation and power production at the ankle and knee joints from the gastrocnemii muscles and suggests an important participation of biarticular mechanisms to the necessary absorption of the centre of mass energy at the beginning of stance. During this phase, the knee joint undergoes a flexion (i.e., lengthening of the vasti muscle-tendon units), thus a part of the energy transferred from ankle to the knee joint will be absorbed by the voluminous monoarticular vasti muscle-tendon units.

The potential of the ankle-to-knee joint energy transfer was not different between the predictable and the adapted drop-like walking perturbations. Considering that the weighted activation of the gastrocnemii muscles was higher in the phase of energy transfer from the ankle to the knee joint during the unpredictable trials, we can argue in favour of a greater contribution of biarticular mechanisms to the absorption of body energy during the unpredictable perturbations, where beneficial mechanisms to control stability modulated by prediction are not available. It is well known that prior experience and knowledge of an expected perturbation provides an opportunity to proactively plan the initiation of the perturbation and modify subsequent responses (Patla 2003; Bierbaum et al. 2011). Experience-based feedforward control reduces the consequences of locomotor perturbations and facilitates a robust and stable gait (Bierbaum et al. 2010; Bohm et al. 2012; Moreno Catalá, Woitalla, and Arampatzis 2016). There was a significant modification in the ankle and knee joint angles of the ipsilateral (perturbed) leg at touch-down after the perturbation (i.e., more ankle plantar- and knee flexion) for the adapted drop-like walking perturbations compared to the unpredictable without prior experience perturbations. In addition, the time from plate-drop to take-off of the contralateral leg was longer in the adapted perturbations (adapted: 123±48 ms, unpredictable: 63±26 ms, *p*<0.001). Due to the longer contact time of the contralateral leg with the ground after the perturbation, the downward movement of the body can be resisted, reducing the consequences of the perturbation and the need for energy absorption via biarticular mechanisms. During the knee-to-ankle joint energy transfer phase (30 to 80% of stance), the activation of the gastrocnemii muscles during the drop-like perturbations was also quite high (on average 50–60% of maximum). The active state of the gastrocnemii muscles suggests that biarticular mechanisms were also involved in the push-off phase. In this phase, the knee joint was extended, which means that the vasti muscle-tendon units were shortening. Due to the shortening of the vasti muscle-tendon units, both contractile work and elastic strain energy recoil, i.e. energy stored in the tendinous structures of the vasti muscles during the first part of stance, can be transferred to the ankle joint. The elastic strain energy stored in the tendinous structures of the vasti may have originated from the absorption of the body's kinetic energy as well as from the absorption of the energy transferred from the ankle to the knee joint via the gastrocnemii muscles in the first part of stance. This scenario shows that the transfer of energy from the ankle to the knee joint and vice versa via the biarticular gastrocnemii muscles could reflect an exchange of energy between elastic tissues (Prilutsky and Zatsiorsky 1994; Arampatzis et al. 2023).

It is worth noting that the ankle-to-knee and knee-to-ankle joint energy transfer potentials are metrics that indicate the possibility, not the magnitude, of energy transfer. Quantifying the magnitude of energy transfer between the ankle and knee joints requires the calculation of the mechanical power of the gastrocnemii muscles at the two joints (Prilutsky, Herzog, and Leonard 1996; Arampatzis et al. 2023; Kharazi et al. 2023). In unperturbed walking, the very low activation of the gastrocnemii muscles in the first 20% of stance suggests negligible involvement of the ankle-to-knee joint energy transfer mechanism. In the phase of simultaneous energy absorption and production (i.e., anti-phase fluctuations at the ankle and knee joints), the increased activation of the gastrocnemii muscles predicts an involvement of biarticularity. A recent study (Kharazi et al. 2023) investigating the mechanical power of the gastrocnemii at the ankle and knee joints found very little energy transfer from the ankle to the knee joint at the preferred walking speed, but an important amount of simultaneous energy absorption and production at both joints via the gastrocnemii muscles. This example shows that by measuring simple kinematic data of the ankle and knee joints and the EMG activity of the gastrocnemii muscles, we can provide information about the involvement of the biarticular mechanisms of the gastrocnemii for energy transfer during gait. Irrespective of muscle activation, a possible active insufficiency of the gastrocnemii muscles may affect the actual energy transfer between the two joints. Insufficiency of the gastrocnemii muscles during maximal plantarflexion contractions has been reported for highly flexed knee positions, i.e., 60° knee angle (Hof and Van den Berg 1977). It has also been reported that the EMG activity of the biarticular GM during maximal voluntary plantar flexion contractions decreased at highly flexed knee joint positions (up to 110°) due to a critical force-length potential of the triceps surae muscles, indicating some effects of a possible achieved insufficiency at these knee joint angles (Arampatzis et al. 2006). For the unpredictable trip-like perturbations, the average knee joint angles were 146 ±3° and 161 ±2° during the ankle-to-knee and knee-to-ankle joint energy transfer potential phases, respectively, and 170 ±2° and 164 ±5° for the adapted perturbations. Similarly, for the unpredictable drop-like perturbations, the average knee joint angles were 163 ±2° and 160 ±3° during the ankle-to-knee and knee-to-ankle joint energy transfer potential phases, respectively, and 163 ±4° and 166 ±3° for the adapted drop-like perturbations. These values indicate no active insufficiency of the gastrocnemii muscles in both the trip-like and drop-like perturbations. We used the same cut-off frequency for filtering the kinematic data between the trip-like and drop-like perturbations, despite the different sampling frequency (120 Hz for the trip-like and 250 Hz for the drop-like), to avoid differences in the excluded frequencies in the data between the two experiments. However, we performed a sensitivity analysis using cut-off filters from 10 to 14 Hz in 1.0 Hz steps and determined the ankle-to-knee and knee-to-ankle joint energy transfer potentials. For the unpredictable trip-like perturbations, the ankle-to-knee joint energy transfer potential ranged from 0.181 ±0.084 to 0.185 ±0.087 and the knee-to-ankle joint energy transfer potential from 0.245 ±0.126 to 0.259 ±0.127. For the adapted trip-like perturbations, the ankle-to-knee joint energy transfer potential ranged from 0.143 ±0.117 to 0.158 ±0.119 and the knee-to-ankle joint energy transfer potential from 0.231 ±0.100 to 0.246 ±0.118. For the unpredictable drop-like perturbations, the ankle-to-knee joint energy transfer potential ranged from 0.264 ±0.052 to 0.267 ±0.0.054 and the knee-to-ankle joint energy transfer potential from 0.337 ±0.108 to 0.348 ±0.106. For the adapted drop-like perturbations, the ankle-to-knee joint energy transfer potential ranged from 0.292 ±0.072 to 0.303 ±0.073 and the knee-to-ankle joint energy transfer potential from 0.343 ±0.116 to 0.350 ±0.115. The sensitivity analysis shows a negligible effect of the chosen cut-off frequency in the trip-like and drop-like perturbations.

In conclusion, the potential for energy transfer between the ankle and knee joint via the biarticular gastrocnemii muscles showed a relevant increase during both trip-like (1.6-fold) and drop-like (2.5-fold) gait perturbations compared to unperturbed walking. This modulation was consistent during the unpredictable without prior experience and adapted with experience walking perturbations indicating a crucial participation of biarticular mechanisms to counteract locomotor disturbances. Furthermore, the high activation of the gastrocnemii muscles in the phase of energy transfer from the ankle to the knee joint during the drop-like perturbations indicates an important involvement of biarticular mechanisms to the necessary absorption of the centre of mass energy at the beginning of the stance. Finally, the increased potential for energy transfer from the knee to the ankle joint, together with the high activation of the gastrocnemii muscles during the push-off phase, suggests a contribution of the voluminous monoarticular vasti muscles to the mechanical power and work required at the ankle joint. This knowledge may be useful in the design of training interventions for fall prevention (i.e., including exercises with in-phase fluctuations at the ankle and knee joints), the construction of bioinspired exoskeletons for customized assistance (Wade, Lichtwark, and Farris 2024), and the development of biarticular actuators in legged robots (Sharbafi et al. 2016).

## Data Availability

The original data of the study can be accessed here (10.6084/m9.figshare.26716510.v1).

## Declarations

**Ethical aproval**

All authors approved this publication and agreed to be held accountable for the work performed therein.
